# A genome-wide screen identifies a single β-defensin gene cluster in the chicken: implications for the origin and evolution of mammalian defensins

**DOI:** 10.1186/1471-2164-5-56

**Published:** 2004-08-13

**Authors:** Yanjing Xiao, Austin L Hughes, Junko Ando, Yoichi Matsuda, Jan-Fang Cheng, Donald Skinner-Noble, Guolong Zhang

**Affiliations:** 1 Department of Animal Science, Oklahoma State University, Stillwater, OK 74078, USA; 2 Department of Biological Sciences, University of South Carolina, Columbia, SC 29208, USA; 3 Center for Advanced Science and Technology, Hokkaido University, Sapporo 060-0810, Japan; 4 Department of Genome Sciences, Lawrence Berkeley National Laboratory, Berkeley, CA 94720, USA

## Abstract

**Background:**

Defensins comprise a large family of cationic antimicrobial peptides that are characterized by the presence of a conserved cysteine-rich defensin motif. Based on the spacing pattern of cysteines, these defensins are broadly divided into five groups, namely plant, invertebrate, α-, β-, and θ-defensins, with the last three groups being mostly found in mammalian species. However, the evolutionary relationships among these five groups of defensins remain controversial.

**Results:**

Following a comprehensive screen, here we report that the chicken genome encodes a total of 13 different β-defensins but with no other groups of defensins being discovered. These chicken β-defensin genes, designated as *Gallinacin 1–13*, are clustered densely within a 86-Kb distance on the chromosome 3q3.5-q3.7. The deduced peptides vary from 63 to 104 amino acid residues in length sharing the characteristic defensin motif. Based on the tissue expression pattern, 13 β-defensin genes can be divided into two subgroups with *Gallinacin 1–7 *being predominantly expressed in bone marrow and the respiratory tract and the remaining genes being restricted to liver and the urogenital tract. Comparative analysis of the defensin clusters among chicken, mouse, and human suggested that vertebrate defensins have evolved from a single β-defensin-like gene, which has undergone rapid duplication, diversification, and translocation in various vertebrate lineages during evolution.

**Conclusions:**

We conclude that the chicken genome encodes only β-defensin sequences and that all mammalian defensins are evolved from a common β-defensin-like ancestor. The α-defensins arose from β-defensins by gene duplication, which may have occurred after the divergence of mammals from other vertebrates, and θ-defensins have arisen from α-defensins specific to the primate lineage. Further analysis of these defensins in different vertebrate lineages will shed light on the mechanisms of host defense and evolution of innate immunity.

## Background

Defensins constitute a large family of small, cysteine-rich, cationic peptides that are capable of killing a broad spectrum of pathogens, including various bacteria, fungi, and certain enveloped viruses [1-5]. These peptides play a critical role in host defense and disease resistance by protecting the hosts against infections. Transgenic mice expressing human enteric defensin *HD5 *are fully protected against the doses of *Salmonella typhimurium *that are otherwise lethal to the wide-type mice [6]. Conversely, mice deficient in the matrilysin gene, which is responsible for activating enteric defensins, become more susceptible to oral infection with *S. typhimurium *[7].

Defensins have been identified in species ranging from plants, insects to animals and humans [1-5]. Characterized by the presence of 6–8 cysteine residues in relatively defined positions, all defensins are structurally related in that they form 3–4 intramolecular disulfide bonds and 2–3 antiparallel β-sheets with or without an α-helix. Based on the spacing pattern of cysteines, these peptides are broadly divided into five groups; namely plant, invertebrate, α-, β-, and θ-defensins [1-5]. Alignment of all known defensin sequences revealed the consensus defensin motif of each group as follows: plant defensin: C-X_8–11_-C-X_3–5_-C-X_3_-C-X_9–12_-C-X_4–11_-C-X_1_-C-X_3_-C; invertebrate defensin: C-X_5–16_-C-X_3_-C-X_9–10_-C-X_4–7_-C-X_1_-C; α-defensin: C-X_1_-C-X_3–4_-C-X_9_-C-X_6–10_-C-C; and β-defensin: C-X_4–8_-C-X_3–5_-C-X_9–13_-C-X_4–7_-C-C. The α- and β-defensins are unique to vertebrate animals with α-defensins only being found in rodents and primates, while β-defensins are present in all mammalian species investigated [1-3]. On the other hand, θ-defensins have only been found in certain primates as a result of posttranslational ligation of two α-defensin-like sequences [8-10]. A pseudogene for θ-defensin is also present in humans [11].

Analysis of human and mouse genomes indicated that β-defensins form 4–5 distinct clusters on different chromosomes with each cluster consisting of multiple defensin genes [12]. Interestingly, the single mammalian α-defensin locus is located on a β-defensin cluster with θ-defensins residing in the center of α-defensins [12]. Studies with mammalian defensins suggested a rapid duplication followed by positive selection and diversification within each group [13-18]. However, the evolutionary relationships among three groups of mammalian defensins and among plant, invertebrate, and mammalian defensins remain controversial. Similarity in spatial structure and biological functions favors the notion that all mammalian defensins are evolutionarily related [19], although a phylogenetic analysis suggested a closer relationship between β- and insect defensins than between α- and β-defensins [16].

Existence of a large number of expressed sequence tag (EST) sequences and recent completion of chicken genome sequencing at a 6.6× coverage [20] provided a timely opportunity to discover a complete repertoire of defensin-related sequences in birds for studying the evolutionary relationship between invertebrate and mammalian defensins. Here we report identification of a single β-defensin cluster that is composed of 13 genes located on the chicken chromosome 3q3.5-q3.7. Evolutionary and comparative analyses of these chicken β-defensins with mammalian homologues strongly suggested that all mammalian defensins have evolved from a common β-defensin-like ancestor, which has undergone rapid duplication, positive diversifying selection, and chromosomal translocations, thereby giving rising to multiple gene clusters on different chromosomal regions.

## Results and Discussion

### Discovery of novel chicken defensins

To identify novel defensin genes in the chicken, all five groups of known defensin-like peptide sequences from plants, invertebrates, and vertebrates were first queried individually against the translated chicken nonredundant (NR), EST, high throughput genomic sequence (HTGS), and whole-genome shortgun sequence (WGS) databases in the GenBank by using the TBLASTN program[21]. All potential hits were then examined manually for the presence of the characteristic cysteine motifs. For every novel defensin identified, additional iterative BLAST searches were performed until no more novel sequences could be found. In addition to three known chicken β-defensins (*Gal 1–3*) [22,23], nine novel putative sequences, namely *Gal 4–12*, have been found in the EST database with at least two hits for each, and such sequences have also been confirmed in genomic sequences (Table 1). Because of the fact that mammalian defensins tend to form clusters [12,14,15,18], all chicken HTGS and WGS sequences containing defensin sequences were also retrieved from GenBank, translated into six open reading frames, and manually curated. As a result, an additional putative β-defensin, *Gal13*, was identified in several genomic clones (Table 1). The open reading frame of *Gal13 *was predicted by GENSCAN [24] and confirmed by directly sequencing of RT-PCR product amplified from chicken kidney.

**Table 1:** Identification of chicken β-defensins

Gene	EST^1,2^	HTGS	WGS	Gene Size (bp)^3^						
				
				E 1	I 1	E 2	I 2	E 3	I 3	E 4
Gal1	BX260462	AC110874	AADN01058097	70	972	88	482	127	496	217
Gal2	BX540940	AC110874	AADN01058097	66	1113	143	183	121	674	204
Gal3		AC110874	AADN01058097 AADN01058096			53	980	109	1180	215
Gal4	BU451960		AADN01058096			136	461	127	117	141
Gal5	BU389548		AADN01058096			290	445	127	355	187
Gal6	CF251501	AC110874	AADN01058097 AADN01058098	52	704	86	705	130	249	234
Gal7	CF251115	AC110874	AADN01058098	50	656	86	201	130	234	248
Gal8	BU242665	AC110874	AADN01058098	71	915	91	259	134	706	494
Gal9	BX270804	AC110874	AADN01058098			220	1592	130	781	343
Gal10	AW198592	AC110874	AADN01058099			118	268	133	1719	381
Gal11	BM440069	AC110874	AADN01058101			63	966	129	1001	460
Gal12	BX257296	AC110874	AADN01058102			84	396	420		
Gal13		AC110874	AADN01058102			61	1016	118	3322	91

^1 ^Abbreviations: *EST*, expressed sequence tag; *HTGS*, high throughput genomic sequence; *WGS*, whole-genome shortgun sequence; *E*, exon;*I*, intron. ^2 ^One EST sequence entry is given only for the exemplary purpose. In each case, more than two independent EST sequences have been found, except for *Gal3 *and *Gal13*, both of which have no EST sequences. *Gal3 *was found through homology cloning [23], and *Gal13 *was predicted by us from the genomic sequence. ^3 ^All *Gal *genes are predicted to consist of four exons separated by three introns, except for *Gal12*, whose last two exons are fused together. The absence of additional sequence information at the 5'-untranslated regions of the cDNA sequences prevented prediction of the sizes of first exon and intron for *Gal 3–5 *and *Gal 9–13 *genes.

No other sequence containing β-defensin-like six-cysteine motif has been found in NR, EST or genomic databases, suggesting that 13 *Gal *genes constitute the entire repertoire of the β-defensin family encoded in the chicken genome. Although it is highly unlikely, we could not rule out the possibility that additional defensin-related genes with distant homology might be uncovered in the chicken by different computational search methods such as the use of Hidden Markov models [12,15]. It is noted that none of other groups of defensins have been discovered in the chicken, indicating that plant, invertebrate, α-, and θ-defensins are absent in the chicken lineage.

**Figure 1:**
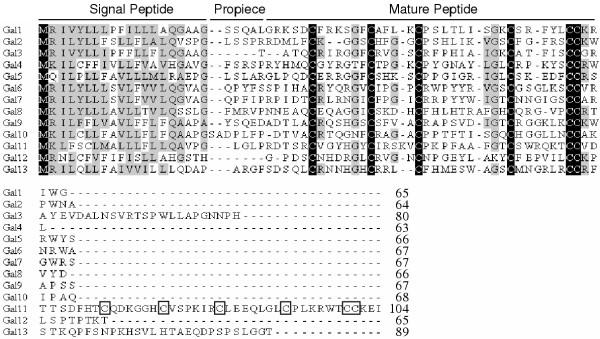
**Multiple sequence alignment of chicken β-defensins. **The intervening region between signal and mature peptide sequence is the short propiece. The conserved residues are shaded. Also shown is the length of each peptide. Notice the six-cysteine defensin motif is highly conserved. The six cysteines in the second tandem copy of the defensin motif in Gal11 are boxed.

Similar to Gal 1–3, 10 novel β-defensins, deduced from either EST or genomic sequences, vary from 63 to 104 amino acid residues in length. Alignment of these peptides revealed a conservation of the signal sequence at the N-terminus and the characteristic six-cysteine defensin motif at the C-terminus (Figure 1). Consistent with the fact that all β-defensins are a group of secreted molecules in response to infections, the signal sequences of all chicken defensins are hydrophobic and rich in leucines. In addition, the mature C-terminal sequences are all positively charged due to the presence of excess arginines and lysines. Interestingly, Gal11 contains two tandem, but highly divergent, copies of the six-cysteine motif at the C-terminus, and is the only defensin having such sequences. Functional significance for existence of such two defensin motifs remains to be studied.

### Evolutionary analysis of vertebrate β-defensins

**Figure 2:**
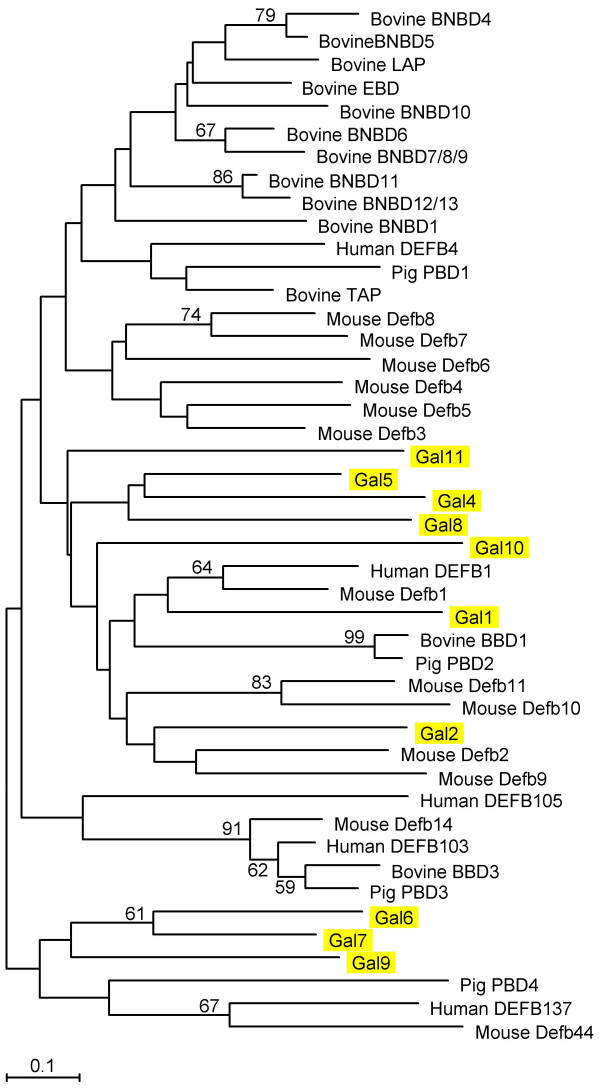
**Phylogenetic relationship of vertebrate β-defensins. **The tree was constructed by the neighbor-joining method and the reliability of each branch was assessed by using 1000 bootstrap replications. Numbers on the branches indicate the percentage of 1000 bootstrap samples supporting the branch. Only branches supported by a bootstrap value of at least 50% are indicated. Chicken β-defensins are highlighted in yellow. Abbreviations: *BNBD*, bovine neutrophil β-defensin; *LAP*, lingual antimicrobial peptide; *EBD*, enteric β-defensin; *TAP*, tracheal antimicrobial peptide; *PBD*, porcine β-defensin; *DEFB/Defb*, β-defensin; *Gal*, Gallinacin; *GAPDH*, glyceraldehyde-3-phosphate dehydrogenase.

Phylogenetic analysis of vertebrate β-defensins showed that chicken defensins clustered with various different groups of mammalian β-defensins (Figure 2). However, the bootstrap support for these patterns was very weak (less than 50% in all cases). The clustering of certain chicken β-defensins with mammalian homologues suggests that major subfamilies of β-defensins arose before the last common ancestor of birds and mammals, estimated to have occurred about 310 million years ago [25]. This in turn implies that some duplication of β-defensin genes must have taken place before the divergence of birds and mammals. The apparent lack of α-defensins in the chicken and other non-mammalian species (G. Zhang, unpublished data) suggests that α-defensins may have evolved after mammals diverged from other vertebrates.

Comparison of the numbers of synonymous and nonsynonymous nucleotide substitutions provides a powerful test of the hypothesis that positive Darwinian selection has acted to favor changes at the amino acid level [26]. This approach has previously been applied to both α- and β-defensins of mammals and has revealed positive selection acting on the mature defensin but not on other regions of the gene [16,17]. In the comparison of the chicken β-defensin sequences, synonymous sites were saturated with changes or nearly so, making it impossible to test the hypothesis of positive selection in every case. In pairwise comparisons among all sequences, mean p_S _in the propeptide region was 0.551 ± 0.036 (S.E.), while mean p_N _was 0.369 ± 0.040. In the mature defensin region, mean p_S _was 0.673 ± 0.027, while mean p_N _was 0.534 ± 0.051. Mean p_N _in the mature defensin was significantly greater than that in the propeptide (z-test; P < 0.05), indicating lesser functional constraint on the amino acid sequence of the former. The high mean p_S _shows that chicken β-defensin genes have not duplicated recently, unlike β-defensin genes of the bovine [16]. In the comparison between the most closely related pair of sequences (*Gal6 *and *Gal7*), mean p_S _in the mature defensin was 0.221 ± 0.082, while mean p_N _was 0.331 ± 0.076. While these values are not significantly different at the 5% level, the fact that p_N _was higher than p_S _suggested that positive selection may have acted to diversify the mature defensin region between these two genes.

### Genomic organization and chromosomal localization of the chicken β-defensin gene cluster

**Figure 3:**
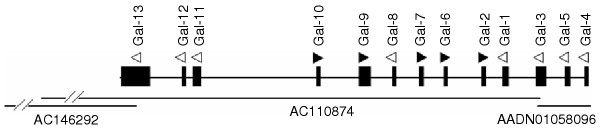
**Genomic organization of the chicken β-defensin gene cluster. **The horizontal lines at the bottom represent the three overlapping genomic clones that were used to assemble the continuous, gap-free contig. The position of each gene is represented by a solid vertical bar and the width of each bar is proportional to the size of each gene. The direction of transcription is indicated by the triangle above each gene. The genes with solid triangles are transcribed in the direction opposite to the ones with open triangles. Slanted lines refer to the sequences omitted. Note that the three fragments of AC110874 sequence have been re-ordered and the gaps have been filled following alignment with AC146292.

**Figure 4:**
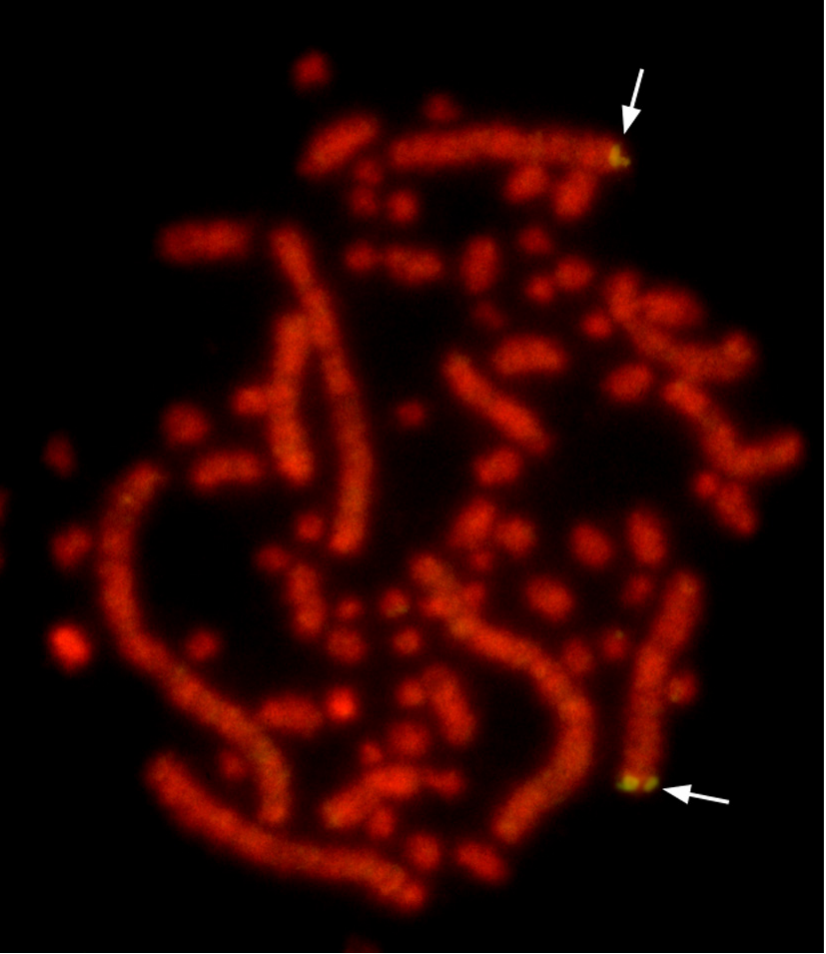
**Chromosomal localization of the chicken β-defensin gene cluster by fluorescence in situ hybridization. **The BAC clone TAM31-54I4, which harbors 11 *Gal *genes, was mapped to chicken chromosome 3q3.5-q3.7. Arrows indicate the hybridization signals.

Searching through HTGS database led to identification of two overlapping bacterial artificial chromosome (BAC) sequences, TAM31-54I5 (accession no. AC110874) and CH261-162O9 (accession no. AC146292), both of which were sequenced and deposited earlier by one of us (J.F. Chen). Alignment of these two sequences allowed to re-order three DNA fragments in AC110874 and to construct a continuous, gap-free genomic contig that includes 11 *Gal *genes except for *Gal4 *and *Gal5*. Later search of chicken WGS sequences released on February 29, 2004 confirmed the order of the genomic contig that we assembled and also revealed the locations of two remaining genes, *Gal4 *and *Gal5*, both of which reside on a WGS (accession no. AADN01058096) that overlaps with AC110874 (Figure 3). The position and orientation of each *Gal *gene were obtained by comparing its cDNA with the assembled DNA sequence. As shown in Figure 3, all 13 *Gal *genes were clustered densely within a distance of 86.0 Kb on the genome. It was also confirmed by aligning such a contig with the chicken genome assembly, in which 13 *Gal *genes are located on six WGS contigs (Table 1) of chromosome 3 that are only ~3.3 Mb from the distal end. Consistent with this, the *Gal *gene cluster was physically mapped to the tip of chicken chromosome 3 at the region of q3.5-q3.7 by fluorescence in situ hybridization (FISH) using the TAM31-54I5 BAC DNA as probe (Figure 4).

Comparing the cDNA with genomic sequences also revealed the structure of each *Gal *gene. Unlike most mammalian β-defensin genes, which primarily consist of two exons and one intron, the *Gal *genes were found to be composed of four short exons separated by three introns with variable lengths ranging from 117 bp to 3,322 bp (Table 1). *Gal12 *is an exception, in which the last two exons have been fused together. While the first exon of the *Gal *genes encodes 5'-untranslated region (UTR) and the majority of the last exon encodes 3'-UTR as well as a few C-terminal amino acids, two internal exons resemble mammalian β-defensin genes in that one exon encodes the signal and pro-sequence and the other encodes the mature sequence with six-cysteine motif [19,27-29]. Apparently, the first two and the last two exons of the *Gal *genes have joined together during the evolution as a result of exon shuffling, which occurred in many other evolutionarily conserved gene families [30], including invertebrate defensins [5]. The fusion of defensin exons in mammals is presumably adaptive because it allows a faster mobilization of such host defense molecules to better cope with invading microbes.

### Tissue expression patterns of chicken β-defensins

**Figure 5:**
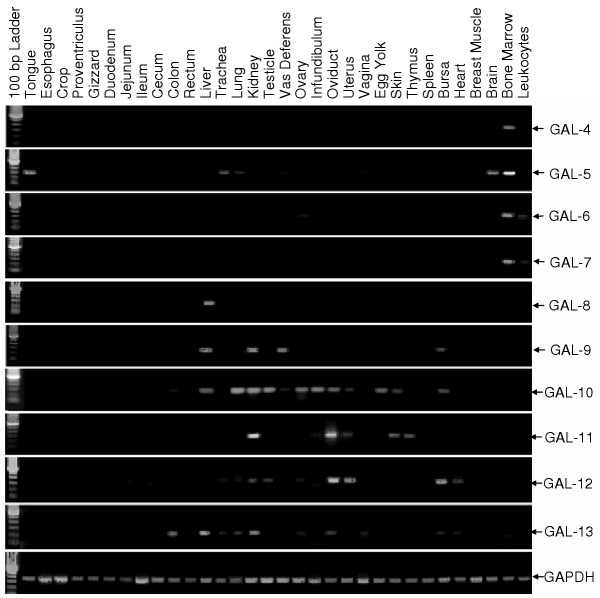
**Tissue expression patterns of 10 novel chicken β-defensins by RT-PCR. **See Materials and Methods for details. The number of PCR cycles was optimized for each gene, and the specificity of each PCR product was confirmed by sequencing. The house-keeping gene, GAPDH, was used for normalization of the template input.

It has been shown that *Gal1 *and *Gal2 *are expressed in bone marrow and lung, while *Gal3 *is more preferentially expressed in bone marrow, tongue, trachea, and bursa of Fabricius [23]. To study the tissue expression patterns of novel *Gal *genes that we identified, RT-PCR was performed with a panel of 32 different chicken tissues. Similar to *Gal 1–3*, *Gal 4–7 *are highly restricted to bone marrow cells with *Gal5 *also expressed in tongue, trachea, lung, and brain at lower levels (Figure 5). By contrast, the six remaining genes, *Gal 8–13*, were not found in bone marrow, but instead in liver, kidney, testicle, ovary, and male and female reproductive tracts (Figure 5). These results clearly suggested that all chicken β-defensin genes can be divided into two subgroups. Seven genes (*Gal 1–7*) are predominantly expressed in bone marrow and the respiratory tract, whereas the other six genes (*Gal 8–13*) are more restricted to liver and the urogenital tract. However, the functional significance and transcriptional regulatory mechanisms of these genes during inflammation and infection remain to be investigated.

### Comparative analysis of chicken and mammalian β-defensin gene clusters

**Figure 6:**
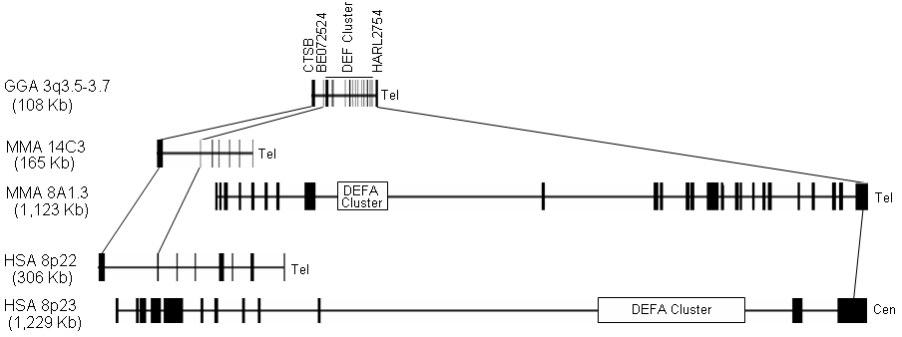
**Comparative analysis of defensin clusters among the chicken, mouse, and human. **The gene clusters were drawn proportionally according to their sizes. Each vertical line/bar represents the position of a gene, and the width of each line/bar is proportional to the size of each gene. Three highly conserved genes (*CTSB*, BE072524, and *HARL2754*) surrounding the defensin clusters in the chicken, mouse, and human were connected by solid lines. The position of the α-defensin locus (*DEFA*) was indicated as an open square. Note that the human θ-defensin pseudogene resides in the *DEFA *locus. The positions and orders of defensin genes in human and mouse were drawn based on the genome assemblies released in July 2003 and October 2003, respectively. Abbreviations: *GGA*, chicken chromosome; *MMA*, mouse chromosome; *HSA*, human chromosome; *Tel*, telomere; *Cen*, centromere.

To study the origin and evolution of mammalian defensins, a comparative analysis of β-defensin gene clusters in the chicken, mouse, and human was performed by employing additional, more phylogenetically conserved gene markers surrounding the defensin clusters. As shown in Figure 6, two genes, *CTSB *(Cathepsin B, accession no. NP_680093) and a human EST sequence (accession no. BE072524) immediately located centromeric to chicken defensins, were also found to be conserved in the defensin gene clusters on human chromosome 8p22 and mouse chromosome 14C3. Similarly, another gene, *HARL2754 *(accession no. XP_372011) that is 6-Kb telemetric to *Gal4 *is also conserved in another defensin cluster in human (8p23) or mouse (8A1.3) (Figure 6).

These results strongly suggested that all vertebrate β-defensins are evolved from a single gene. This conclusion is further supported by the fact that there are three highly similar β-defensin-like sequences present in the largely finished zebrafish genome (G. Zhang, unpublished data). In addition, a group of homologous β-defensin-like sequences, namely crotamine and myotoxins, have been found in several *Crotalus *snakes [31], which are presumably derived from a single ancestral gene. The appearance of multiple β-defensin gene clusters on different chromosomal regions in mammalian species [12] is apparently a result of rapid gene duplication, positive diversifying selection, and chromosomal translocation following divergence of mammals from other vertebrate lineages.

In addition to the structural conservation between β-defensin-like sequences in the rattlesnake and mammals [32], a growing body of evidence suggests that their functions appear to be largely conserved in that both are capable of interacting negatively-charged lipid membranes followed by formation of ion channels or pores [32-34]. It is noteworthy that the conservation of Cathepsin B (CTSB) adjacent to β-defensins is perhaps not surprising, given the recent finding that cathepsins are involved in the cleavage and inactivation of β-defensins [35].

## Conclusions

We have showed that chicken genome encodes a total of 13 different β-defensin genes clustered densely within a 86-Kb distance on the chromosome 3q3.5-q3.7, but with no α-defensin genes. These peptides exhibit homology to different subgroups of mammalian β-defensins-, consistent with the hypothesis that α-defensins and β-defensins arose by gene duplication after the divergence of birds and mammals. The θ-defensins are specific to primates; and thus appear to have arisen from α-defensins by gene duplication specific to the primate lineage. Apparently, the evolution of defensins is rapid and driven by duplication and positive diversifying selection. Collectively, this study represents the first large-scale detailed investigation of defensins in non-mammalian vertebrates. There is no doubt that further analysis of these defensin genes will lead to a better understanding of host defense mechanisms and evolution of innate immunity.

## Methods

### Computational search for novel chicken defensins

To identify novel defensins in the chicken, all known cysteine-containing defensin-like peptide sequences discovered in plants, invertebrates, birds, and mammals were individually queried against the translated chicken NR, EST, HTGS, and WGS databases in the GenBank by using the TBLASTN program [21] with default settings on the NCBI web site [36]. All potential hits were then examined for the presence of the characteristic defensin motif. For every novel defensin identified, additional iterative BLAST searches were performed until no more novel sequences could be revealed. Because mammalian defensins tend to form clusters [12,14,15,18], all chicken genomic sequences containing defensin sequences were also retrieved from the GenBank and translated into six open reading frames and curated manually for the presence of the defensin motif in order to discover potential sequences with distant homology.

### Alignment and phylogenetic analysis of chicken β-defensins

Multiple sequence alignment was constructed by using the ClustalW program (version 1.82) [37]. A phylogenetic tree of amino acid sequences of mature β-defensins was constructed by the neighbor-joining method [38]. So that a comparable data set would be used for all pairwise comparisons, any site at which the alignment postulated a gap in any sequence was excluded from the analysis. To maximize the number of sites available for analysis, certain sequences with large deletions were excluded from the analysis. Because the sequences were very short (25 aligned sites), no correction for multiple hits was applied. The reliability of clustering patterns within the tree was assessed by bootstrapping; 1000 bootstrap pseudo-samples were used. The proportion of synonymous nucleotide differences per synonymous site (p_S_) and the proportion of nonsynonymous nucleotide differences per nonsynonymous site (p_N_) were estimated by the method of Nei and Gojobori [26]. Again, no correction for multiple hits was applied because a small number of sites were examined.

### Assembly of the chicken β-defensin gene cluster

To generate a continuous defensin gene cluster, the HTGS and WGS sequences containing the putative defensin genes were retrieved from the GenBank, aligned to generate a longer contig, which was confirmed later by searching through the assembled chicken genome released on February 29, 2004, by using the BLAT program [39] under the UCSC Genome Browser web site [40]. The relative positions, orientations, and structural organizations of individual genes were determined by comparing its cDNA sequence to the continuous genomic contig that we assembled.

### Chromosome localization of the chicken β-defensin gene cluster

Fluorescence in situ hybridization (FISH) was used for chromosomal assignment of the chicken β-defensin gene cluster by using the BAC clone TAM31-54I4 as probe, which harbors 11 *Gal *genes. Metaphase chromosome speads were prepared from mitogen-stimulated chicken splenocyte culture as we described [41,42]. The BAC clone was labeled by nick translation with biotin 16-dUTP (Roche Diagnostics), hybridized to metaphase chromosome DNA, followed by detection with FITC-labeled avidin (Roche Diagnostics) and staining with propidium iodide to simultaneously induce the R-banding.

### RT-PCR analysis of the tissue expression patterns of chicken β-defensins

Total RNA was extracted with Trizol (Invitrogen) from a total of 32 different tissues from healthy, 2-month-old chickens (see Figure 5). A total of 4 μg RNA from each tissue were reverse transcribed with random hexamers and Superscript II reverse transcriptase by using a first-strand cDNA synthesis kit (Invitrogen) according to the instructions. The subsequent PCR was carried out with 1/40 of the first-strand cDNA and gene-specific primers for each β-defensin and glyceraldehyde-3-phosphate dehydrogenase (GAPDH) as described [28,43]. Every pair of primers were designed to locate on different exons to aid in distinguishing PCR products amplified from cDNA vs. genomic DNA (Table 2). The PCR program used was: 94°C denaturation for 2 min, followed by different cycles of 94°C denaturation for 20 sec, 55°C annealing for 20 sec, and 72°C extension for 40 sec, followed by a final extension at 72°C for 5 min. The number of PCR cycle was optimized for each gene to ensure linear amplification (Table 2). A half of the PCR products were analyzed by electrophoresis on 1.2% agarose gels containing 0.5 μg/ml ethidium bromide. The specificity of each PCR product was confirmed by cloning of the PCR product into T/A cloning vector, followed by sequencing of the recombinant plasmid.

**Table 2:** Primer sequences used for RT-PCR analysis of novel chicken β-defensins

Gene	Primer Sequence		Product Size (bp)		Cycles Used
		
	Sense	Antisense	cDNA	Genomic	
Gal4	CATCTCAGTGTCGTTTCTCTGC	ACAATGGTTCCCCAAATCCAAC	321	899	36
Gal5	CTGCCAGCAAGAAAGGAACCTG	TGAACGTGAAGGGACATCAGAG	300	1100	36
Gal6	AGGATTTCACATCCCAGCCGTG	CAGGAGAAGCCAGTGAGTCATC	249	1203	36
Gal7	CTGCTGTCTGTCCTCTTTGTGG	CATTTGGTAGATGCAGGAAGGA	230	665	35
Gal8	ACAGTGTGAGCAGGCAGGAGGGA	CTCTTCTGTTCAGCCTTTGGTG	261	967	35
Gal9	GCAAAGGCTATTCCACAGCAG	AGCATTTCAGCTTCCCACCAC	211	1802	33
Gal10	TGGGGCACGCAGTCCACAAC	ATCAGCTCCTCAAGGCAGTG	298	2285	33
Gal11	ACTGCATCCGTTCCAAAGTCTG	TCGGGCAGCTTCTCTACAAC	301	1299	33
Gal12	CCCAGCAGGACCAAAGCAATG	GTGAATCCACAGCCAATGAGAG	335	731	36
Gal13	CATCGTTGTCATTCTCCTCCTC	ACTTGCAGCGTGTGGGAGTTG	175	4514	50
GAPDH	GCACGCCATCACTATCTTCC	CATCCACCGTCTTCTGTGTG	356	876	30

## Note added in proof

Following submission of this manuscript, Lynn *et al. *reported independently discovery of seven novel chicken β-defensins in the chicken EST database by using homology search strategies [44]. Consistent with our conclusion, they also revealed occurrence of positive selection particularly in the mature region of chicken β-defensins following evolutionary analysis. Moreover, albeit the use of a different nomenclature, they confirmed that the expressions of *Gal 4–7 *are primarily in bone marrow, while other genes are more restricted to liver and the genitourinary tract.

## List of abbreviations

Abbreviations: Gal, Gallinacin; NR, nonredundant; EST, expressed sequence tag; HTGS, high throughput genomic sequence; WGS, whole-genome shortgun sequence; BAC, bacterial artificial chromosome; FISH, fluorescence in situ hybridization; UTR, untranslated region; GAPDH, glyceraldehyde-3-phosphate dehydrogenase.

## Authors' contributions

YX carried out the tissue collection, RT-PCR analysis of tissue expression patterns, and drafted the manuscript. ALH carried out the phylogenetic and molecular evolutionary analyses. JA and YM carried out the fluorescence in situ hybridization. JFC carried out the sequencing of two chicken defensin-containing BAC clones. DSN participated in tissue collection and preparation. GZ conceived of the study, carried out all computational analyses and annotation, drafted the manuscript, and participated in its design and coordination. All authors read and approved the final manuscript.
